# Statistical Inference Methods for Two Crossing Survival Curves: A Comparison of Methods

**DOI:** 10.1371/journal.pone.0116774

**Published:** 2015-01-23

**Authors:** Huimin Li, Dong Han, Yawen Hou, Huilin Chen, Zheng Chen

**Affiliations:** 1 Department of Biostatistics, School of Public Health and Tropical Medicine, Southern Medical University, Guangzhou, China; 2 Department of Statistics, College of Economics, Jinan University, Guangzhou, China; Indiana University Bloomington, UNITED STATES

## Abstract

A common problem that is encountered in medical applications is the overall homogeneity of survival distributions when two survival curves cross each other. A survey demonstrated that under this condition, which was an obvious violation of the assumption of proportional hazard rates, the log-rank test was still used in 70% of studies. Several statistical methods have been proposed to solve this problem. However, in many applications, it is difficult to specify the types of survival differences and choose an appropriate method prior to analysis. Thus, we conducted an extensive series of Monte Carlo simulations to investigate the power and type I error rate of these procedures under various patterns of crossing survival curves with different censoring rates and distribution parameters. Our objective was to evaluate the strengths and weaknesses of tests in different situations and for various censoring rates and to recommend an appropriate test that will not fail for a wide range of applications. Simulation studies demonstrated that adaptive Neyman’s smooth tests and the two-stage procedure offer higher power and greater stability than other methods when the survival distributions cross at early, middle or late times. Even for proportional hazards, both methods maintain acceptable power compared with the log-rank test. In terms of the type I error rate, Renyi and Cramér—von Mises tests are relatively conservative, whereas the statistics of the Lin-Xu test exhibit apparent inflation as the censoring rate increases. Other tests produce results close to the nominal 0.05 level. In conclusion, adaptive Neyman’s smooth tests and the two-stage procedure are found to be the most stable and feasible approaches for a variety of situations and censoring rates. Therefore, they are applicable to a wider spectrum of alternatives compared with other tests.

## Introduction

In clinical studies, the task of comparing the overall equality of two survival distributions with censored observations is a key element in survival analysis. It is well known that the commonly used log-rank test has optimum power under the assumption of proportional hazard rates. However, this assumption is often violated, especially when two survival curves cross each other. It has been demonstrated that the log-rank test may lose power in this situation [1–5]. The phenomenon of crossing survival curves is common [6–15] when a treatment may offer a short-term benefit but does not provide long-term advantages. In a study conducted by Mok [12], the log-rank test was used to evaluate the effects of two treatments even when the two survival curves crossed. However, Seruga et al. [16] and Bouliotis et al. [17] have observed that if the curves cross, there is a clear violation of the proportional-hazards assumption, and they have suggested the use of other statistical methods that do not rely on these assumptions. Kristiansen [18] has reviewed all publications in five notable journals and identified 175 studies concerning survival analysis, among which crossing survival curves were present in 47% of the studies. Of those studies in which crossing survival curves were present, the log-rank test was performed in 70% of the tests, and only 31% of them reported testing for proportional hazards. Using the log-rank test under conditions of non-proportional hazards may lead to misleading results, therefore, Bouliotis et al. [17] have stated that “*there is a need in the clinical community to clarify methods that are appropriate when survival curves cross*.”

In the literature, there are a fair number of statistical methodologies for addressing the crossing-curves problem. Gill [19] has proposed a supremum version of the weighted log-rank test, the Renyi test. Fleming et al. [20] have developed the modified Kolmogorov—Smirnov test for the correct comparison of crossing survival curves. Koziol [21] and Schumacher [22] have generalized the Cramér—von Mises test to censored data. The above methods are based on the integrated difference of the Nelson—Aalen cumulative hazard functions.

Another category of methods is the weighted Kaplan—Meier tests based on the integrated weighted difference of two Kaplan—Meier estimators. Pepe and Fleming [23] have demonstrated that weighted Kaplan—Meier tests perform better than the log-rank test in the crossing hazards scenario. To address the problem of choosing a suitable weight function in advance, some versatile tests have been developed based on the maximum version of the weighted Kaplan—Meier test [24] or the combination of the Fleming—Harrington test [25]. Lin and Wang [4] have proposed a test statistic that measures the squared differences at each time point. Lin and Xu [1] have designed a specialized method to compare two crossing survival curves based on the absolute difference between the area under the two curves. Qiu and Sheng [2] have suggested a two-stage procedure in which the log-rank test serves as the first stage and a proposed procedure for addressing the crossing hazard rates is applied in the second stage. Kraus [26] has constructed a class of Neyman’s smooth tests based on the concept of Neyman’s embedding and a data-driven strategy.

For most of the procedures mentioned above, the performances of the corresponding test statistics have been evaluated by considering several survival scenarios. Simulation studies have demonstrated that the tests developed by Lin and Wang [4] and by Lin and Xu [1] perform better than do the log-rank and Wilcoxon tests in the case of crossing survival curves. Tubert-Bitter et al. [27] have considered early, middle and late crossings of the survival curves. Moreover, Liu et al. [3] have conducted a comprehensive simulation study of three different patterns of crossing hazard rates and have demonstrated that some of weighted log-rank tests (log-rank, Gehan—Wilcoxon and Peto—Peto) lose power compared with methods that are specifically designed to address the problem of crossing hazard rates.

As mentioned above, few simulation studies have accounted for the scenario in which two survival curves cross at some particular location, which is a focal point to be discussed in our study. Most studies have considered only a few of the new testing approaches and have not evaluated the statistical properties of these new approaches under high censoring rates. Therefore, we performed Monte Carlo simulations to investigate whether the power of these methods was robust under various configurations of crossing survival curves and to determine the behavior of these methods with variations in censoring rates. Our objective is to evaluate the strengths and weaknesses of the tests for a variety of situations and censoring rates and to recommend an appropriate test that will not fail for a wide range of applications. The article is structured as follows: In the following section, we briefly introduce several existing methods. The statistical properties of these methods in a variety of situations are presented in Section 3. All methods are illustrated using three examples in Section 4. Finally, in Section 5, we summarize our findings and present a general discussion.

## Methods

### Assumptions and notations

Our study focuses on the comparison of methods for testing the omnibus hypothesis:

$$H_{0}:S_{1}\left(t\right)=S_{2}\left(t\right)\;\text{for}\;\text{all}\;t,\;H_{1}:S_{1}\left(t\right)\neq S_{2}\left(t\right)\;,$$

for two survival functions *S*_1_(*t*) and *S_2_* (*t*) that cross each other. If two survival functions cross each other, then the corresponding hazard rates also cross each other [3].

Consider two censored samples of sample size *N*_i_ (*i* = 1, 2), and let *t*_1_ < … < *t_j_* < … < *t_τ_* (*j* = 1, 2, …, *τ*) be the distinct failure times in the pooled sample, where *t_τ_* is the latest event time at which two groups contain at least one subject at risk. The Kaplan—Meier estimator is defined as $\hat{S}(t)=1$ for *t* < *t*_1_ and $\hat{S}(t)=\prod_{t_{j}\leq t}\left(1-d_{j}/n_{j}\right)$ for *t* ≥ *t*_1_, where *d_j_* and *n_j_* denote the number of observed failures and the number of individuals who are at risk at time *t_j_*, respectively.

### Existing methods

We first consider methods constructed based on the difference between the Nelson—Aalen cumulative hazard functions. It is well known that when different weight functions are selected, the weighted log-rank test can generate a variety of methods, such as the log-rank (LR) test, the Gehan—Wilcoxon [28] (GW) test, the Tarone-Ware [29] (TW) test and a general class of Fleming-Harrington [30–31] (*G^ργ^*) tests with the weight function ${\left[\hat{S}(t-)\right]}^{\rho }{\left[1-\hat{S}(t-)\right]}^{\gamma }(\rho \geq 0,\gamma \geq 0)$. Even so, when crossing survival curves appear, early positive differences between the two hazard rates are canceled out by later differences of the opposite sign, which may cause some weighted log-rank tests to lose power [3]. As a result, considerable efforts have been directed toward improving the sensitivity of testing approaches in such cases. Instead of taking the sum over all time points, Gill’s [19] Renyi (RY) test improves the testing power by replacing the numerator of the weighted log-rank statistic with a supremum. Fleming et al. [20] have also modified the classical Kolmogorov—Smirnov test and generalized it for use with arbitrarily right-censored data. Analogous to the Renyi test, the modified Kolmogorov—Smirnov statistic (MKS) is also a supremum approach, but this test is more sensitive to apparent differences in survival distributions at a single point in time. Moreover, Schumacher [22] has generalized the classical Cramér—von Mises test for application to right-censored data, modifying the test to be based on the integrated squared difference between two estimated hazard rates [31]. There are two types of Cramér—von Mises statistics: one is asymptotic to a standard Brownian motion process (CVM1), and the other converges to a Brownian bridge process (CVM2) [32].

In another class of methodologies, which includes those that are constructed in terms of the difference between two estimated survival functions, Pepe and Fleming [23] have introduced a class of statistics that constitute the Weighted Kaplan—Meier test (WKM), which is based on the integrated weighted difference between Kaplan—Meier estimators. Similar to the weighted log-rank test, a prior misspecification of the weight function may decrease the power of the WKM test. Of greater concern is the fact that a series of trials using different test statistics may lead to a multiple comparison problem and to the inflation of the overall type I error rate. These shortcomings of the WKM test motivated Shen and Cai [24] to propose a maximum WKM test (MKM) using a set of weighted Kaplan—Meier statistics {*V*_1_, *V*_2_, … *V*_m_}, where the weight functions are generated from all possible combinations of *ρ* = 0, 1, 2 and *γ* = 0, 1, 2 in the function ${\left[\hat{S}(t-)\right]}^{\rho }{\left[1-\hat{S}(t-)\right]}^{\gamma }(\rho \geq 0,\gamma \geq 0)$. Meanwhile, with appropriate justification, the type I error rate is well controlled in a multivariate Gaussian distribution.

In recent years, many scholars have proposed new approaches for detection in a wide range of circumstances. Lee [25] has developed three versatile tests based on the combination of two Fleming—Harrington statistics using *ρ* = 1, *γ* = 0 and *ρ* = 0, *γ* = 1. These combined tests are based on (1) the absolute value of the average of the two statistics (SHL1), (2) the average of their absolute values (SHL2) and (3) the maximum of their absolute values (SHL3). Using the method proposed by Yang et al. [33], the critical values and *P* values of these latter two versatile tests can be obtained. Lin and Wang [4] (LW) have proposed a new approach to comparing the overall homogeneity of survival curves by measuring the squared differences between the number of observed failures and the number of expected failures over time. Lin and Xu [1] have suggested a new method for comparing survival distributions based on the absolute difference between the area under two survival curves; here, the one-sided and two-sided test are denoted by LX1 and LX2, respectively. With the intent of addressing all possible alternatives, Qiu and Sheng [2] have proposed a two-stage procedure (TS). In stage one, a weighted log-rank test, such as the log-rank test, is conducted. If a rejection of the null hypotheses is obtained in stage one, then the entire procedure is terminated; otherwise, the result suggests that either the two hazard rates may be identical or they may cross each other, but these two scenarios cannot be distinguished by the stage-one procedure. Thus, the method proceeds to stage two, in which the basic idea is to choose a particular weight for the weighted log-rank test that changes signs before and after a potential crossing point. Because the two test statistics in two individual stages are proved to be asymptotically independent of each other under *H*_0_, then the p-value of the entire test can be redefined. Kraus’s test [26] is based on the concept of Neyman’s embedding combined with Schwarz’s selection rule. The null hypothesis *h*_1_(*t*) = *h*_2_(*t*) is tested against a model in which the hazard ratio of the two survival distributions is expressed by *d* smooth functions. By constructing the partial likelihood score statistic *T_d_*, *d* fixed-dimensional smooth tests are defined. However, one would not know how to specify the number of smooth functions for the test prior to analysis. Therefore, the author introduced Schwarz’s selection rule and proposed a more reasonable data-driven test statistic, *T*_s_. When the number of high priority basis functions *d*_0_ is specified, the data-driven test statistics of nested subsets or of all subsets are asymptotically *χ* ^2^ distributed with different degrees of freedom. The author has demonstrated that in practice, a test with a relatively small fixed dimension *d* or with nested subsets of smaller *d*_0_ often performs very well. See Kraus [26] for more details. Based on the results of the simulation studies cited by Kraus, we selected a fixed-dimensional test statistic *T_d_* (*d* = 4) (NY1) and a data-driven test statistic *T_s_^nested^* (*d* = 8, *d*_0_ = 0) (NY2) for further simulations because these statistics exhibited the greatest stability of discrimination power among all evaluated parameters. For consistency with the original text, both tests were performed using 2000 permutations. A brief summary of the methods investigated in this study is listed in S1 Table.

## Simulation

### Simulation design

To assess the performance of the tests mentioned above, we conducted Monte Carlo simulations for various random censoring rates (0%, 20%, 40% and 60%) and the following situations: (A) two groups with proportional hazard rates, (B) two crossing survival curves with the crossing point located at *S*(*t*)>0.6, (C) two survival curves crossing at *S*(*t*) = 0.4~0.6 and (D) two survival curves crossing at *S*(*t*) = 0.2~0.4. Meanwhile, we considered equal sample sizes (*N*_1_ = *N*_2_ = 20, 50, 100) and unequal sample sizes (*N*_1_ = 20, *N*_2_ = 30; *N*_1_ = 40, *N*_2_ = 80; *N*_1_ = 20, *N*_2_ = 50; *N*_1_ = 50, *N*_2_ = 100) in each group. For each sample size, 5000 iterations were performed for each situation and censoring rate. The exact power and size of these test statistics were estimated by determining the proportion of samples for which the null hypothesis was rejected at the *α* = 0.05 significance level.

**Figure 1:**
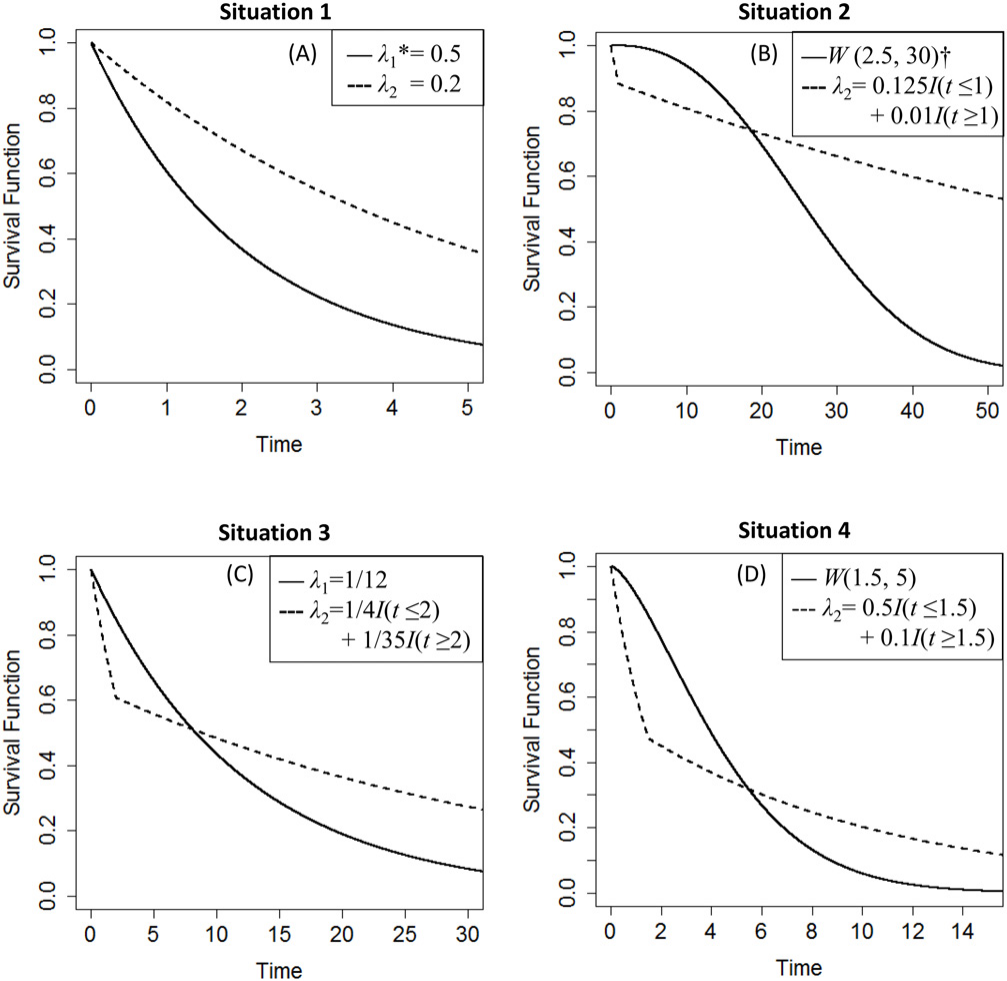
Survival configurations for simulation of power. * *λ*_i_ denotes the hazard function of *S*_i_(*t*), a piecewise exponential distribution. † W(*η*, *θ*) denotes a Weibull distribution having *S*(*t*) = exp{(-*t* / *θ*)^*η*^}, where *η*>0 is the shape parameter, *θ*>0 is the scale parameter.

A simulation of time-to-event data was performed based on a randomly censored model [34–35]. We generate individual failure times *X* following the survival functions (A)-(D) in Fig. 1; (B)-(D) are defined to be approximately equal to the real data described in Example and (A) a proportional hazards model for reference. Next, the censoring time *Cr* in two samples was generated from uniform distributions *U* (0, *a*) and *U* (0, *b*), where varying the values of *a* and *b* may result in censoring rates of approximately 20%, 40% or 60% in the two samples. Each individual was assigned an observed survival time *T* = min(*X*, *Cr*) and an event indicator *δ* = I[*X*≤*Cr*]. Because the lifetime *X* followed different distributions in each group, it was necessary for the values of *a* and *b* to be unequal to keep the average censoring rates in each group approximately equal to the given censoring rates.

### Estimation of statistical power and type I error

**Situation 1**. When two survival curves have proportional hazard functions (see Fig. 1A), the powers of various test statistics rise with increasing sample size. By contrast, for a particular sample size, the powers of the tests decline with increasing censoring rate (see Table 1). As expected, the LR test demonstrates the optimal power in this situation, followed by the SHL1, SHL2 and SHL3 tests. When each sample size is equal to 20 and when the censoring rate is increased to 60%, the power of each test becomes less than 40%, a relatively low level. However, when each sample size is greater than or equal to 50 while the censoring rate is simultaneously no more than 20%, all tests demonstrate powers above 80%. In general, the LR, SHL1, SHL2 and SHL3 tests are far superior to the other tests under the assumption of proportional hazard rates.

**Table 1:** Power of test procedures for Situation 1.

**N**	**CENR**	**LR**	**GW**	**TW**	**G01**	**G10**	**G11**	**RY**	**MKS**	**CVM1**	**CVM2**	**WKM**	**MKM**	**SHL1**	**SHL2**	**SHL3**	**LW**	**LX1**	**LX2**	**TS**	**NY1**	**NY2**
(20, 20)	0%	0.7794	0.6736	0.7346	0.6966	0.6736	0.7054	0.6986	0.6454	0.5900	0.5014	0.6696	0.6496	0.7866	0.7860	0.7436	0.5384	0.7192	0.6350	0.6862	0.5492	0.5316
	20%	0.6730	0.5792	0.6362	0.5814	0.6022	0.6064	0.6004	0.5772	0.4744	0.4022	0.5652	0.5562	0.6794	0.6792	0.6508	0.4596	0.6044	0.5176	0.5760	0.4810	0.4486
	40%	0.5334	0.4650	0.5064	0.4450	0.4968	0.4738	0.4636	0.4198	0.3638	0.3060	0.4620	0.4532	0.5378	0.5378	0.5124	0.3552	0.4976	0.4298	0.4414	0.3852	0.3652
	60%	0.3878	0.3474	0.3710	0.3230	0.3760	0.3340	0.3266	0.2350	0.2482	0.1996	0.3320	0.3378	0.3828	0.3828	0.3744	0.2616	0.3448	0.3032	0.2992	0.2962	0.2760
(50, 50)	0%	0.9938	0.9734	0.9870	0.9712	0.9734	0.9800	0.9876	0.9622	0.9690	0.9284	0.9876	0.9824	0.9942	0.9942	0.9876	0.9266	0.9874	0.9732	0.9844	0.9514	0.9728
	20%	0.9740	0.9386	0.9652	0.9240	0.9494	0.9530	0.9640	0.9378	0.9180	0.8590	0.9640	0.9580	0.9760	0.9760	0.9632	0.8554	0.8894	0.8522	0.9526	0.8888	0.9322
	40%	0.9134	0.8568	0.8942	0.8090	0.8862	0.8606	0.8840	0.8452	0.8064	0.7206	0.8848	0.8834	0.9064	0.9064	0.8932	0.7152	0.7266	0.6720	0.8594	0.7638	0.8246
	60%	0.7512	0.6928	0.7276	0.6280	0.7322	0.6626	0.7042	0.6030	0.6052	0.5256	0.6962	0.7092	0.7346	0.7346	0.7304	0.5198	0.5228	0.4522	0.6624	0.5740	0.6348
(100, 100)	0%	1.0000	0.9994	1.0000	0.9998	0.9994	0.9998	1.0000	0.9994	0.9994	0.9984	1.0000	1.0000	1.0000	1.0000	1.0000	0.9992	1.0000	1.0000	1.0000	0.9998	1.0000
	20%	0.9998	0.9980	0.9998	0.9982	0.9990	0.9996	0.9998	0.9986	0.9980	0.9954	0.9998	0.9998	0.9998	0.9998	0.9998	0.9942	0.9860	0.9772	0.9998	0.9970	0.9996
	40%	0.9978	0.9918	0.9960	0.9830	0.9956	0.9922	0.9960	0.9906	0.9872	0.9636	0.9950	0.9958	0.9974	0.9974	0.9960	0.9546	0.8600	0.8262	0.9942	0.9774	0.9936
	60%	0.9638	0.9428	0.9598	0.8972	0.9614	0.9268	0.9538	0.9188	0.9022	0.8408	0.9468	0.9548	0.9592	0.9592	0.9552	0.8174	0.6978	0.6486	0.9408	0.8770	0.9398
(20, 30)	0%	0.8724	0.7886	0.8352	0.7910	0.7886	0.8116	0.8114	0.7548	0.6376	0.5274	0.7600	0.7468	0.8802	0.8800	0.8460	0.7886	0.8456	0.7820	0.7992	0.6846	0.6882
	20%	0.7700	0.6884	0.7388	0.6776	0.7096	0.7094	0.7092	0.6398	0.5050	0.4084	0.6528	0.6434	0.7766	0.7762	0.7588	0.6932	0.6902	0.6228	0.6860	0.6046	0.5918
	40%	0.6388	0.5688	0.6084	0.5236	0.5978	0.5576	0.5758	0.4866	0.3804	0.3002	0.5370	0.5424	0.6336	0.6336	0.6234	0.5508	0.5374	0.4762	0.5424	0.4960	0.4826
	60%	0.4638	0.4276	0.4502	0.3880	0.4492	0.4046	0.4146	0.2794	0.2404	0.1800	0.4074	0.4186	0.4628	0.4628	0.4650	0.4144	0.3684	0.3244	0.3812	0.3924	0.3722
(20, 50)	0%	0.9286	0.8658	0.9012	0.8564	0.8658	0.8848	0.8898	0.8092	0.6734	0.5586	0.8276	0.8136	0.9338	0.9338	0.9108	0.9066	0.9302	0.8956	0.8790	0.7950	0.8064
	20%	0.8470	0.7750	0.8160	0.7434	0.7918	0.7928	0.7998	0.7076	0.5424	0.4256	0.7334	0.7264	0.8496	0.8494	0.8326	0.8224	0.7300	0.6876	0.7780	0.7178	0.7086
	40%	0.7186	0.6706	0.6998	0.5952	0.6926	0.6330	0.6746	0.5572	0.3888	0.2902	0.6256	0.6322	0.7044	0.7042	0.7062	0.6844	0.5620	0.5128	0.6366	0.5914	0.5888
	60%	0.5540	0.5186	0.5390	0.4594	0.5404	0.4826	0.5052	0.3284	0.2214	0.1448	0.4736	0.4942	0.5468	0.5468	0.5486	0.5298	0.3728	0.3248	0.4696	0.4746	0.4706
(40, 80)	0%	0.9970	0.9844	0.9922	0.9798	0.9844	0.9916	0.9936	0.9722	0.9644	0.9218	0.9894	0.9874	0.9972	0.9972	0.9942	0.9942	0.9952	0.9920	0.9926	0.9736	0.9884
	20%	0.9792	0.9570	0.9716	0.9270	0.9626	0.9564	0.9692	0.9390	0.9014	0.8240	0.9636	0.9568	0.9780	0.9780	0.9724	0.9676	0.8752	0.8458	0.9600	0.9184	0.9490
	40%	0.9240	0.8888	0.9122	0.8224	0.9068	0.8646	0.9016	0.8430	0.7650	0.6712	0.8864	0.8862	0.9162	0.9162	0.9108	0.9000	0.6930	0.6422	0.8804	0.8208	0.8616
	60%	0.7802	0.7454	0.7694	0.6450	0.7714	0.6800	0.7502	0.5972	0.5368	0.4394	0.7282	0.7448	0.7646	0.7646	0.7678	0.7446	0.4858	0.4192	0.7074	0.6496	0.6940
(50, 100)	0%	0.9992	0.9958	0.9984	0.9916	0.9958	0.9976	0.9986	0.9932	0.9902	0.9734	0.9982	0.9972	0.9996	0.9994	0.9988	0.9988	0.9992	0.9982	0.9984	0.9930	0.9980
	20%	0.9946	0.9830	0.9920	0.9630	0.9866	0.9856	0.9910	0.9770	0.9576	0.9148	0.9884	0.9878	0.9950	0.9950	0.9910	0.9914	0.9206	0.8976	0.9896	0.9720	0.9848
	40%	0.9662	0.9438	0.9586	0.8960	0.9570	0.9312	0.9558	0.9210	0.8732	0.7858	0.9500	0.9512	0.9618	0.9618	0.9604	0.9508	0.7348	0.6920	0.9432	0.8982	0.9326
	60%	0.8560	0.8218	0.8460	0.7448	0.8480	0.7762	0.8326	0.7188	0.6580	0.5608	0.8100	0.8306	0.8420	0.8420	0.8446	0.8294	0.5496	0.4896	0.8018	0.7430	0.7910

The notations represented are listed as follows. N: sample size of two groups; CENR: censoring rate; LR: Log-rank; G01: Fleming-Harrington test ρ = 0, γ = 1; G10: Fleming-Harrington test ρ = 1, γ = 0; G11: Fleming-Harrington test ρ = 1, γ = 1; GW: Gehan-Wilcoxon; TW: Tarone-Ware; SHL1: |Z1+Z2|/2; SHL2: (|Z1|+|Z2|)/2; SHL3: max(|Z1|, |Z2|); MKS: Modified Kolmogorov-Smirnov test; CVM1: Cramér-von Mises test based on Brownian motion; CVM2: Cramér-von Mises test based on Brownian bridge; WKM: Weighted Kaplan-Meier test; MKM: Maximum of the WKM tests; LW: Lin and Wang’s test; LX1: Lin and Xu’s one-sided test; LX2: Lin and Xu’s two-sided test; TS: Two-stage test; NY1: Neyman’s test d = 4, not data-driven; NY2: Neyman’s test with d = 8, d_0_ = 0, data-driven and nested.

**Situation 2**. For the early (*S*(*t*)>0.6) crossing of the survival curves (Fig. 1B), Table 2 summarizes the results of the power estimations. When the sample size of each group is not less than 50, we note that the powers of all tests gradually decrease with increasing censoring rate. By contrast, for small sample sizes (*N*_1_ = *N*_2_ = 20), the powers of most tests (except GW, CVM1 and WKM) increase unexpectedly when the censoring rate reaches 60%. High censoring rates appears to have considerable influence on the power of the G10, TW, GW and WKM tests. When the censoring rate is increased to 60%, these four statistics suffer significant loss of power (decreasing to 8.14%, 7.66%, 3.26% and 2.24%, respectively). The weight function of the WKM test is associated with the Kaplan—Meier estimator ${\hat{S}}_{i}(t)$, which can be very unstable in the presence of heavy censoring. To remedy this instability, the statistic down-weights the contribution of S^1(t)−S^(t)2 if the censoring is heavy, and as a result, the WKM test loses power in such a situation. In contrast, the TW, GW and G10 tests assign a greater weight to earlier failure times, causing these tests to be insensitive to differences at later times. Overall, the G01, G11, SHL3, LW, LX, TS, NY1 and NY2 tests perform better than do the others. Among them, the NY1 and NY2 tests are the most stable, and except for small (*N*_1_ = *N*_2_ = 20) sample sizes, the powers of both statistics exceed 80% for various combinations of sample sizes and censoring rates.

**Table 2:** Power of test procedures for Situation 2.

**N**	**CENR**	**LR**	**GW**	**TW**	**G01**	**G10**	**G11**	**RY**	**MKS**	**CVM1**	**CVM2**	**WKM**	**MKM**	**SHL1**	**SHL2**	**SHL3**	**LW**	**LX1**	**LX2**	**TS**	**NY1**	**NY2**
(20, 20)	0%	0.9270	0.5510	0.7674	0.9992	0.5510	0.9928	0.8780	0.9784	0.3800	0.1882	0.2682	0.7070	0.9672	0.9826	0.9988	0.9924	0.9828	0.9532	0.9190	0.9980	0.9972
	20%	0.8042	0.3360	0.5628	0.9966	0.4150	0.9780	0.7096	0.9312	0.1968	0.0974	0.1668	0.5242	0.9142	0.9334	0.9922	0.9782	0.9496	0.9176	0.8000	0.9920	0.9884
	40%	0.0814	0.0658	0.0634	0.3988	0.0652	0.3626	0.0830	0.0842	0.0734	0.1048	0.0914	0.1160	0.1864	0.1870	0.3424	0.4798	0.2092	0.2742	0.2152	0.5016	0.5454
	60%	0.1946	0.0596	0.0940	0.6752	0.1012	0.6236	0.1440	0.2874	0.0426	0.0724	0.0484	0.1484	0.3770	0.3806	0.6030	0.6240	0.5434	0.4750	0.4850	0.7186	0.7504
(50, 50)	0%	0.9998	0.8956	0.9868	1.0000	0.8956	1.0000	0.9998	1.0000	0.9524	0.9326	0.7850	0.9996	1.0000	1.0000	1.0000	1.0000	1.0000	1.0000	1.0000	1.0000	1.0000
	20%	0.9934	0.6638	0.9126	1.0000	0.7738	1.0000	0.9932	1.0000	0.7318	0.7494	0.5730	0.9890	0.9992	1.0000	1.0000	1.0000	0.9986	0.9978	0.9998	1.0000	1.0000
	40%	0.9056	0.2946	0.6170	1.0000	0.5374	0.9994	0.8838	0.9948	0.3238	0.4856	0.2652	0.8930	0.9850	0.9964	1.0000	1.0000	0.9882	0.9826	0.9762	1.0000	1.0000
	60%	0.4134	0.0644	0.1534	0.9672	0.1772	0.9476	0.4128	0.7406	0.1298	0.4026	0.0532	0.4940	0.7448	0.7954	0.9532	0.9654	0.8362	0.7472	0.9190	0.9900	0.9872
(100, 100)	0%	1.0000	0.9948	1.0000	1.0000	0.9948	1.0000	1.0000	1.0000	1.0000	1.0000	0.9902	1.0000	1.0000	1.0000	1.0000	1.0000	1.0000	1.0000	1.0000	1.0000	1.0000
	20%	1.0000	0.9070	0.9980	1.0000	0.9662	1.0000	1.0000	1.0000	1.0000	0.9998	0.9338	1.0000	1.0000	1.0000	1.0000	1.0000	1.0000	1.0000	1.0000	1.0000	1.0000
	40%	0.9982	0.4784	0.8836	1.0000	0.8114	1.0000	0.9994	1.0000	0.9550	0.9896	0.5840	1.0000	1.0000	1.0000	1.0000	1.0000	1.0000	1.0000	1.0000	1.0000	1.0000
	60%	0.7004	0.0702	0.2328	0.9998	0.2868	0.9990	0.8610	0.9842	0.5832	0.9304	0.0626	0.8944	0.9598	0.9894	0.9998	0.9994	0.9880	0.9726	0.9972	1.0000	1.0000
(20, 30)	0%	0.9676	0.6520	0.8422	1.0000	0.6520	0.9990	0.9404	0.9986	0.3740	0.2352	0.2952	0.7780	0.9918	0.9962	1.0000	0.9998	0.9986	0.9958	0.9704	1.0000	1.0000
	20%	0.8506	0.4050	0.6292	0.9982	0.4880	0.9916	0.7750	0.9650	0.1632	0.1226	0.1672	0.5864	0.9600	0.9692	0.9972	0.9944	0.9676	0.9578	0.8610	0.9982	0.9992
	40%	0.5672	0.1622	0.3260	0.9688	0.2736	0.9418	0.4638	0.7666	0.0684	0.1110	0.0724	0.3404	0.8136	0.8244	0.9570	0.9284	0.9032	0.8678	0.6914	0.9740	0.9780
	60%	0.2000	0.0518	0.0890	0.7332	0.0952	0.6748	0.1506	0.3156	0.0720	0.1700	0.0350	0.1450	0.4382	0.4406	0.6668	0.5812	0.5886	0.5184	0.6472	0.7940	0.8348
(20, 50)	0%	0.9930	0.7658	0.9290	1.0000	0.7658	1.0000	0.9820	1.0000	0.3556	0.3898	0.3150	0.8450	0.9994	1.0000	1.0000	1.0000	1.0000	1.0000	0.9942	1.0000	1.0000
	20%	0.9140	0.4504	0.7016	1.0000	0.5414	0.9988	0.8494	0.9882	0.1318	0.2630	0.1644	0.6552	0.9894	0.9920	0.9996	0.9942	0.9682	0.9620	0.9168	1.0000	1.0000
	40%	0.5898	0.1582	0.3268	0.9850	0.2704	0.9672	0.4694	0.8204	0.0712	0.2736	0.0614	0.3596	0.8780	0.8834	0.9754	0.8838	0.9152	0.8838	0.6878	0.9832	0.9900
	60%	0.1828	0.0326	0.0766	0.7878	0.0814	0.7238	0.1348	0.3372	0.1372	0.3670	0.0224	0.1362	0.4680	0.4700	0.7352	0.4292	0.5996	0.5338	0.7560	0.8406	0.8928
(40, 80)	0%	1.0000	0.9488	0.9960	1.0000	0.9488	1.0000	0.9998	1.0000	0.9588	0.9666	0.7638	0.9964	1.0000	1.0000	1.0000	1.0000	1.0000	1.0000	1.0000	1.0000	1.0000
	20%	0.9972	0.7146	0.9366	1.0000	0.8154	1.0000	0.9940	1.0000	0.7018	0.8620	0.5080	0.9786	1.0000	1.0000	1.0000	1.0000	0.9962	0.9934	1.0000	1.0000	1.0000
	40%	0.8850	0.2746	0.5868	0.9998	0.4964	0.9992	0.8454	0.9922	0.3318	0.7178	0.1840	0.8234	0.9884	0.9952	0.9998	0.9968	0.9868	0.9792	0.9648	1.0000	1.0000
	60%	0.3390	0.0410	0.1150	0.9564	0.1334	0.9256	0.3566	0.6884	0.2868	0.7026	0.0348	0.3720	0.7230	0.7566	0.9380	0.7884	0.8022	0.7000	0.9238	0.9892	0.9950
(50, 100)	0%	1.0000	0.9800	0.9998	1.0000	0.9800	1.0000	1.0000	1.0000	0.9950	0.9982	0.8842	1.0000	1.0000	1.0000	1.0000	1.0000	1.0000	1.0000	1.0000	1.0000	1.0000
	20%	0.9996	0.8074	0.9746	1.0000	0.9008	1.0000	0.9998	1.0000	0.9112	0.9718	0.6638	0.9978	1.0000	1.0000	1.0000	1.0000	0.9988	0.9986	1.0000	1.0000	1.0000
	40%	0.9426	0.3282	0.6902	1.0000	0.5916	1.0000	0.9372	0.9986	0.5606	0.8864	0.2594	0.9218	0.9972	0.9992	1.0000	0.9994	0.9954	0.9932	0.9956	1.0000	1.0000
	60%	0.4112	0.0368	0.1246	0.9830	0.1520	0.9676	0.4744	0.8154	0.4080	0.8416	0.0332	0.4712	0.8206	0.8660	0.9734	0.8766	0.8770	0.7958	0.9600	0.9984	0.9994

**Situation 3**. When considering middle (*S*(*t*) = 0.4~0.6) crossing of the survival curves (Fig. 1C), the NY2, NY1, G01 and SHL3 tests, followed by the TS, G11, MKS, SHL2, LW and LX tests, exhibit much better performances than the remaining tests for low and moderate censoring rates. However, as Table 3 demonstrates, when the censoring rates are gradually increased to 60%, the powers of the G01, G11, SHL2, SHL1 and LW tests decrease sharply to 4.58%, 5.14%, 6.70%, 6.70% and 2.60%, respectively. This result implies that these five tests are more likely to be affected by high censoring rates. Meanwhile, tests that are not designed to address the crossing-hazard problem, such as the LR, GW and TW tests, maintain a relatively low power in most cases. After a comprehensive comparison of these tests, it is apparent that NY1, NY2, TS, MKS and SHL3 are most applicable for scenarios with middle crossings of survival curves and with high censoring rates. When each sample size is increased 100, these four tests (NY1, NY2, TS and MKS) maintain powers of greater than 80% for censoring rates between 0% and 60%. When a small sample size (*N*_1_ = *N*_2_ = 20) is considered, NY1 and NY2 perform slightly better at various censoring rates.

**Table 3:** Power of test procedures for Situation 3.

**N**	**CENR**	**LR**	**GW**	**TW**	**G01**	**G10**	**G11**	**RY**	**MKS**	**CVM1**	**CVM2**	**WKM**	**MKM**	**SHL1**	**SHL2**	**SHL3**	**LW**	**LX1**	**LX2**	**TS**	**NY1**	**NY2**
(20, 20)	0%	0.1998	0.0600	0.0942	0.6364	0.0600	0.4258	0.1442	0.3458	0.0480	0.0990	0.0944	0.1568	0.2666	0.3198	0.5548	0.3022	0.3422	0.2316	0.4128	0.5714	0.6268
	20%	0.0968	0.0642	0.0544	0.4524	0.0582	0.2992	0.0980	0.2740	0.0580	0.1282	0.0500	0.1058	0.1604	0.1890	0.3822	0.2464	0.3142	0.2270	0.4398	0.4748	0.5402
	40%	0.0514	0.0968	0.0624	0.2546	0.0738	0.1646	0.0924	0.1822	0.1026	0.1648	0.0476	0.0958	0.0804	0.0850	0.2252	0.1452	0.2272	0.1640	0.4486	0.3890	0.4646
	60%	0.1242	0.2124	0.1710	0.0458	0.1658	0.0568	0.1726	0.1386	0.2090	0.2172	0.1810	0.1964	0.0832	0.0832	0.1270	0.0276	0.1638	0.1324	0.1726	0.1828	0.2154
(50, 50)	0%	0.4452	0.0642	0.1408	0.9512	0.0642	0.7666	0.4590	0.8306	0.1576	0.4122	0.3644	0.5744	0.5368	0.8078	0.9358	0.7506	0.8260	0.7234	0.8640	0.9688	0.9728
	20%	0.1890	0.0860	0.0582	0.8242	0.0676	0.6012	0.3156	0.7134	0.1692	0.4386	0.1290	0.3376	0.3204	0.5708	0.7830	0.6010	0.6760	0.5794	0.8204	0.9154	0.9460
	40%	0.0580	0.1830	0.0818	0.5104	0.1092	0.3160	0.3360	0.5544	0.3042	0.5084	0.0498	0.2002	0.1174	0.2354	0.4964	0.3518	0.3952	0.3036	0.8070	0.8318	0.8828
	60%	0.2460	0.4832	0.3870	0.0470	0.3700	0.0584	0.5108	0.4518	0.5616	0.5896	0.3926	0.4396	0.1286	0.1296	0.3016	0.0260	0.2152	0.1766	0.5604	0.5466	0.5900
(100, 100)	0%	0.7680	0.0672	0.2364	0.9990	0.0672	0.9644	0.9214	0.9932	0.6962	0.9060	0.7816	0.9630	0.8360	0.9946	0.9996	0.9756	0.9950	0.9878	0.9988	1.0000	0.9998
	20%	0.3538	0.1026	0.0666	0.9858	0.0700	0.8748	0.8062	0.9818	0.5518	0.8482	0.3264	0.7508	0.5592	0.9422	0.9848	0.9100	0.9224	0.8848	0.9948	0.9988	0.9996
	40%	0.0670	0.3058	0.1044	0.8170	0.1530	0.5616	0.7686	0.9214	0.6522	0.8534	0.0460	0.3690	0.1904	0.6908	0.8336	0.6730	0.7040	0.5998	0.9896	0.9928	0.9968
	60%	0.4134	0.7816	0.6474	0.0494	0.6170	0.0596	0.8642	0.8276	0.8690	0.8910	0.6578	0.7420	0.1914	0.2154	0.5498	0.0264	0.2774	0.2236	0.8590	0.9014	0.8862
(20, 30)	0%	0.1930	0.0428	0.0726	0.7110	0.0428	0.4636	0.1476	0.4250	0.0562	0.1748	0.0764	0.1418	0.2896	0.3590	0.6236	0.4390	0.4834	0.3708	0.4760	0.6604	0.7442
	20%	0.0854	0.0518	0.0384	0.5036	0.0434	0.3222	0.0962	0.3066	0.0976	0.2254	0.0350	0.0970	0.1688	0.1968	0.4340	0.2948	0.4080	0.3120	0.4940	0.5580	0.6502
	40%	0.0412	0.0944	0.0500	0.2778	0.0616	0.1658	0.1060	0.2154	0.1734	0.2868	0.0410	0.0904	0.0786	0.0868	0.2384	0.1590	0.2668	0.2058	0.4822	0.4520	0.5288
	60%	0.1244	0.2280	0.1854	0.0498	0.1766	0.0602	0.1990	0.1860	0.3254	0.3494	0.2038	0.2126	0.0804	0.0804	0.1358	0.2114	0.1832	0.1468	0.2048	0.1946	0.2478
(20, 50)	0%	0.1900	0.0260	0.0618	0.8012	0.0260	0.5262	0.1470	0.4706	0.0860	0.3018	0.0682	0.1344	0.3276	0.4096	0.7338	0.3890	0.6962	0.5874	0.5662	0.7626	0.8386
	20%	0.0748	0.0286	0.0268	0.5834	0.0232	0.3498	0.0988	0.3446	0.1648	0.3632	0.0336	0.0784	0.1796	0.2132	0.5066	0.2016	0.5306	0.4498	0.5644	0.6486	0.7438
	40%	0.0334	0.0732	0.0344	0.3162	0.0412	0.1746	0.1212	0.2542	0.2850	0.4242	0.0328	0.0710	0.0812	0.0876	0.2682	0.0898	0.3306	0.2662	0.5436	0.5324	0.6198
	60%	0.1074	0.2278	0.1748	0.0558	0.1616	0.0514	0.2118	0.2286	0.4514	0.4846	0.1982	0.2130	0.0670	0.0670	0.1254	0.2794	0.1980	0.1554	0.2144	0.2052	0.2692
(40, 80)	0%	0.3940	0.0358	0.1120	0.9738	0.0358	0.7936	0.4560	0.8578	0.2358	0.5614	0.2952	0.4942	0.5686	0.8280	0.9568	0.7550	0.9258	0.8726	0.8608	0.9816	0.9886
	20%	0.1474	0.0560	0.0406	0.8532	0.0404	0.5750	0.3552	0.7348	0.3126	0.5940	0.0998	0.2572	0.3162	0.5512	0.8048	0.4624	0.7332	0.6656	0.8424	0.9368	0.9640
	40%	0.0418	0.1488	0.0586	0.5148	0.0860	0.2764	0.3866	0.5798	0.4740	0.6438	0.0382	0.1454	0.1100	0.2022	0.4712	0.1536	0.4478	0.3546	0.8290	0.8606	0.9026
	60%	0.2054	0.4752	0.3602	0.0506	0.3306	0.0510	0.5146	0.4994	0.6648	0.6886	0.3746	0.4256	0.1028	0.1032	0.2688	0.5500	0.2518	0.2062	0.5094	0.5352	0.5678
(50, 100)	0%	0.4904	0.0352	0.1184	0.9904	0.0352	0.8736	0.6270	0.9316	0.3702	0.7054	0.4170	0.6700	0.6646	0.9296	0.9842	0.8502	0.9694	0.9412	0.9414	0.9960	0.9966
	20%	0.1642	0.0618	0.0328	0.9190	0.0442	0.6700	0.5002	0.8580	0.4250	0.7132	0.1242	0.3552	0.3706	0.7080	0.8894	0.5480	0.8102	0.7514	0.9160	0.9806	0.9902
	40%	0.0354	0.1916	0.0702	0.5972	0.1036	0.3374	0.5342	0.7226	0.5838	0.7476	0.0384	0.1792	0.1150	0.3068	0.5762	0.1766	0.5190	0.4270	0.9054	0.9298	0.9530
	60%	0.2578	0.5804	0.4544	0.0528	0.4174	0.0656	0.6646	0.6384	0.7736	0.7794	0.4662	0.5308	0.1322	0.1322	0.3452	0.6408	0.2784	0.2234	0.6250	0.6826	0.6896

**Situation 4**. For late (*S*(*t*) = 0.2~0.4) crossing of the survival curves (Fig. 1D), the results are presented in Table 4. For increasing censoring rates, the MKS, TS, NY1 and NY2 tests exhibit gradually decreasing power, whereas the powers of the LR, G10, GW, TW, RY, CVM1, WKM and MKM tests increase, and the other tests exhibit various degrees of fluctuation. However, for small (*N*_1_ = *N*_2_ = 20) and unequal (*N*_1_ = 20, *N*_2_ = 50) sample sizes and for high censoring rates, the NY2, NY1 and TS tests are the most powerful tests, followed by the MKS, SHL3, CVM2, SHL2, MKM, CVM1 and RY tests. When each sample size is equal to 100, the tests listed above demonstrate powers greater than 98% regardless of the censoring rate. Although the LW and LX tests are both specialized methods for detecting differences when the survival curves cross, they exhibit lower power for small (*N*_1_ = *N*_2_ = 20) and unequal (*N*_1_ = 20, *N*_2_ = 30; *N*_1_ = 20, *N*_2_ = 50) sample sizes. In addition, it is evident that the LW test is more likely to be affected by high censoring rates and unbalanced design. Therefore, the NY2, NY1 and TS tests are most robust for late crossings of the survival curves.

**Table 4:** Power of test procedures for Situation 4.

**N**	**CENR**	**LR**	**GW**	**TW**	**G01**	**G10**	**G11**	**RY**	**MKS**	**CVM1**	**CVM2**	**WKM**	**MKM**	**SHL1**	**SHL2**	**SHL3**	**LW**	**LX1**	**LX2**	**TS**	**NY1**	**NY2**
(20, 20)	0%	0.0572	0.2598	0.1292	0.4888	0.2598	0.1744	0.2396	0.5730	0.2670	0.4966	0.1112	0.3880	0.0820	0.4058	0.5684	0.1718	0.3316	0.1864	0.7812	0.7914	0.8644
	20%	0.0764	0.3676	0.2166	0.2716	0.3156	0.1120	0.3068	0.5296	0.3654	0.5494	0.1818	0.4118	0.0712	0.2436	0.4346	0.1222	0.3702	0.2430	0.8082	0.6826	0.7758
	40%	0.2246	0.5276	0.3898	0.0870	0.4234	0.0916	0.4192	0.4722	0.5094	0.6110	0.3586	0.4952	0.1428	0.1630	0.3688	0.0458	0.3138	0.2416	0.6630	0.5360	0.6274
	60%	0.6246	0.6822	0.6688	0.3504	0.6624	0.3942	0.6168	0.4056	0.6004	0.5472	0.6988	0.6770	0.5354	0.5354	0.5852	0.2818	0.3594	0.3304	0.5590	0.4258	0.4624
(50, 50)	0%	0.0588	0.5084	0.1854	0.8922	0.5084	0.3006	0.8006	0.9826	0.7532	0.9496	0.0824	0.8286	0.1088	0.9842	0.9798	0.7162	0.9316	0.8370	0.9908	0.9998	1.0000
	20%	0.0638	0.7220	0.3970	0.6368	0.6236	0.1544	0.8462	0.9640	0.8452	0.9574	0.1810	0.8262	0.0598	0.9182	0.8996	0.4818	0.8430	0.7376	0.9960	0.9948	0.9984
	40%	0.3876	0.9048	0.7484	0.1270	0.7938	0.0924	0.9012	0.9310	0.9286	0.9692	0.6220	0.8922	0.2176	0.5954	0.7836	0.0484	0.6080	0.4872	0.9724	0.9652	0.9798
	60%	0.9466	0.9732	0.9664	0.6116	0.9620	0.7078	0.9626	0.8766	0.9666	0.9592	0.9740	0.9688	0.8796	0.8796	0.9408	0.5086	0.3868	0.3756	0.9514	0.8828	0.9122
(100, 100)	0%	0.0838	0.7794	0.2892	0.9950	0.7794	0.4924	0.9988	1.0000	0.9958	1.0000	0.0690	0.9992	0.1736	1.0000	1.0000	0.9920	0.9996	0.9994	1.0000	1.0000	1.0000
	20%	0.0742	0.9484	0.6486	0.9160	0.8906	0.2338	0.9986	0.9998	0.9966	1.0000	0.2194	0.9942	0.0620	1.0000	0.9996	0.8882	0.9968	0.9890	1.0000	1.0000	1.0000
	40%	0.6248	0.9952	0.9600	0.1966	0.9748	0.1002	0.9988	0.9992	0.9988	0.9996	0.8826	0.9940	0.3414	0.9846	0.9878	0.0580	0.9004	0.8314	0.9994	0.9998	0.9998
	60%	0.9992	0.9998	0.9998	0.8756	0.9998	0.9366	1.0000	0.9978	0.9998	0.9994	0.9998	0.9998	0.9932	0.9932	0.9990	0.7926	0.4188	0.4006	0.9992	0.9980	0.9990
(20, 30)	0%	0.0318	0.2496	0.0952	0.5432	0.2496	0.1536	0.3208	0.6906	0.4344	0.6900	0.0908	0.3926	0.0618	0.4752	0.5932	0.2628	0.5368	0.3542	0.8658	0.8646	0.9252
	20%	0.0490	0.3720	0.1896	0.2974	0.3068	0.0880	0.3698	0.6346	0.5348	0.7244	0.1650	0.4298	0.0468	0.2404	0.4382	0.1458	0.4812	0.3470	0.8658	0.7568	0.8652
	40%	0.2030	0.5574	0.4006	0.0718	0.4400	0.0834	0.4868	0.5838	0.6672	0.7662	0.3670	0.5318	0.1278	0.1434	0.3782	0.2200	0.3588	0.2648	0.7068	0.5698	0.6870
	60%	0.6542	0.7208	0.6998	0.3546	0.6912	0.4224	0.6580	0.5060	0.7600	0.7394	0.7438	0.7164	0.5632	0.5632	0.6122	0.0714	0.3918	0.3706	0.5978	0.3836	0.4718
(20, 50)	0%	0.0110	0.2418	0.0748	0.5922	0.2418	0.1116	0.4398	0.7956	0.6654	0.8644	0.0694	0.4020	0.0400	0.5500	0.6380	0.0934	0.7740	0.6244	0.9494	0.9320	0.9762
	20%	0.0296	0.4008	0.1838	0.3008	0.3236	0.0586	0.4964	0.7518	0.7470	0.8812	0.1530	0.4628	0.0282	0.2500	0.4468	0.0880	0.6382	0.5158	0.9296	0.8382	0.9256
	40%	0.1858	0.6218	0.4424	0.0722	0.4834	0.0686	0.5824	0.7082	0.8334	0.8968	0.3898	0.5934	0.1132	0.1274	0.4014	0.4618	0.4442	0.3420	0.7410	0.6312	0.7624
	60%	0.7210	0.7974	0.7770	0.3880	0.7676	0.4734	0.7416	0.6546	0.8968	0.8816	0.8248	0.8058	0.6292	0.6292	0.6864	0.6170	0.4562	0.4310	0.6558	0.3140	0.4848
(40, 80)	0%	0.0164	0.4896	0.1458	0.8892	0.4896	0.1994	0.8720	0.9880	0.9166	0.9834	0.0530	0.7458	0.0656	0.9896	0.9758	0.3672	0.9804	0.9466	0.9954	0.9994	0.9998
	20%	0.0468	0.7336	0.3786	0.5790	0.6274	0.0862	0.9014	0.9750	0.9480	0.9864	0.1748	0.7940	0.0294	0.9010	0.8722	0.1280	0.8924	0.8210	0.9988	0.9956	0.9998
	40%	0.3488	0.9102	0.7566	0.1034	0.8032	0.0842	0.9292	0.9564	0.9730	0.9872	0.6208	0.8888	0.1898	0.4874	0.7768	0.6470	0.6778	0.5600	0.9672	0.9612	0.9784
	60%	0.9504	0.9800	0.9738	0.6456	0.9704	0.7564	0.9698	0.9330	0.9876	0.9842	0.9826	0.9814	0.8946	0.8946	0.9500	0.6008	0.4408	0.4170	0.9570	0.8456	0.9066
(50, 100)	0%	0.0216	0.5814	0.1802	0.9480	0.5814	0.2400	0.9606	0.9976	0.9694	0.9964	0.0456	0.8600	0.0790	0.9992	0.9956	0.4780	0.9972	0.9904	0.9994	1.0000	1.0000
	20%	0.0498	0.8238	0.4610	0.6854	0.7298	0.0964	0.9678	0.9946	0.9826	0.9962	0.1856	0.8760	0.0362	0.9784	0.9586	0.1442	0.9510	0.9170	1.0000	0.9994	1.0000
	40%	0.4264	0.9570	0.8480	0.1114	0.8848	0.0942	0.9766	0.9884	0.9938	0.9972	0.6954	0.9446	0.2246	0.6916	0.8762	0.7012	0.7718	0.6672	0.9928	0.9948	0.9966
	60%	0.9842	0.9940	0.9920	0.7352	0.9908	0.8416	0.9918	0.9750	0.9970	0.9952	0.9946	0.9942	0.9478	0.9478	0.9848	0.6968	0.4448	0.4296	0.9846	0.9400	0.9606

**Situation 5**. To investigate type I errors, two samples were independently generated from an exponential distribution with a hazard rate of *λ*_1_ = *λ*_2_ = 0.25. Various censoring rates were also considered by setting various uniform distribution parameters. The estimated type I error rates are presented in Table 5. For all combinations of sample sizes and censoring rates, the type I error rates for G01, SHL1, SHL2 and SHL3 are slightly inflated, whereas the RY test is more conservative. Although the CVM1 and CVM2 tests are relatively conservative for small sample sizes (*N*_1_ = *N*_2_ = 20), both statistics gradually approach the nominal level of 0.05 as the sample sizes increase. Note that the type I error rates of the LX1 (one-sided) and LX2 (two-sided) tests appear to increase with increasing censoring rates, increasing from 0.0328 to 0.0868 and from 0.0194 to 0.0606, respectively. When the censoring rate reaches 60%, the type I error rate of the LX1 test exceeds the nominal level for all combinations of sample sizes. In addition to the above tests, all statistics are quite close to the nominal level of 0.05.

**Table 5:** Type I error of test procedures.

**N**	**CENR**	**LR**	**GW**	**TW**	**G01**	**G10**	**G11**	**RY**	**MKS**	**CVM1**	**CVM2**	**WKM**	**MKM**	**SHL1**	**SHL2**	**SHL3**	**LW**	**LX1**	**LX2**	**TS**	**NY1**	**NY2**
(20, 20)	0%	0.0552	0.0506	0.0502	0.0724	0.0506	0.0530	0.0372	0.0472	0.0330	0.0358	0.0386	0.0396	0.0582	0.0582	0.0600	0.0450	0.0406	0.0218	0.0488	0.0518	0.0480
	20%	0.0538	0.0498	0.0480	0.0722	0.0506	0.0524	0.0338	0.0508	0.0328	0.0356	0.0376	0.0388	0.0584	0.0584	0.0598	0.0512	0.0546	0.0322	0.0514	0.0544	0.0542
	40%	0.0496	0.0484	0.0486	0.0618	0.0460	0.0508	0.0348	0.0428	0.0344	0.0370	0.0410	0.0402	0.0508	0.0508	0.0526	0.0434	0.0766	0.0498	0.0458	0.0556	0.0530
	60%	0.0514	0.0496	0.0518	0.0570	0.0516	0.0558	0.0404	0.0392	0.0370	0.0356	0.0508	0.0510	0.0546	0.0546	0.0516	0.0412	0.0844	0.0580	0.0428	0.0502	0.0512
(50, 50)	0%	0.0512	0.0500	0.0520	0.0568	0.0500	0.0512	0.0430	0.0478	0.0444	0.0456	0.0434	0.0444	0.0520	0.0518	0.0554	0.0468	0.0360	0.0206	0.0446	0.0546	0.0472
	20%	0.0540	0.0534	0.0528	0.0608	0.0518	0.0528	0.0440	0.0486	0.0428	0.0478	0.0462	0.0474	0.0550	0.0552	0.0594	0.0486	0.0468	0.0278	0.0500	0.0582	0.0518
	40%	0.0568	0.0542	0.0546	0.0628	0.0558	0.0590	0.0490	0.0510	0.0484	0.0484	0.0520	0.0500	0.0590	0.0590	0.0568	0.0468	0.0660	0.0436	0.0554	0.0524	0.0526
	60%	0.0550	0.0584	0.0560	0.0580	0.0564	0.0574	0.0462	0.0430	0.0514	0.0500	0.0586	0.0558	0.0582	0.0582	0.0580	0.0422	0.0728	0.0482	0.0530	0.0550	0.0528
(100, 100)	0%	0.0512	0.0500	0.0502	0.0570	0.0500	0.0540	0.0466	0.0428	0.0466	0.0460	0.0450	0.0468	0.0522	0.0518	0.0562	0.0512	0.0328	0.0194	0.0472	0.0466	0.0522
	20%	0.0530	0.0470	0.0514	0.0566	0.0474	0.0522	0.0460	0.0474	0.0458	0.0456	0.0480	0.0470	0.0548	0.0544	0.0562	0.0512	0.0382	0.0228	0.0468	0.0492	0.0478
	40%	0.0498	0.0492	0.0504	0.0568	0.0494	0.0514	0.0440	0.0482	0.0454	0.0448	0.0488	0.0484	0.0508	0.0508	0.0512	0.0474	0.0504	0.0318	0.0464	0.0508	0.0520
	60%	0.0482	0.0464	0.0442	0.0488	0.0470	0.0500	0.0406	0.0436	0.0412	0.0438	0.0448	0.0454	0.0474	0.0474	0.0468	0.0428	0.0568	0.0356	0.0506	0.0536	0.0490
(20, 30)	0%	0.0526	0.0488	0.0516	0.0636	0.0488	0.0492	0.0364	0.0528	0.0392	0.0412	0.0402	0.0438	0.0562	0.0556	0.0558	0.0544	0.0394	0.0226	0.0432	0.0496	0.0526
	20%	0.0494	0.0482	0.0494	0.0588	0.0486	0.0452	0.0382	0.0552	0.0404	0.0424	0.0428	0.0432	0.0520	0.0522	0.0566	0.0480	0.0500	0.0282	0.0458	0.0466	0.0466
	40%	0.0502	0.0510	0.0534	0.0568	0.0542	0.0524	0.0416	0.0498	0.0420	0.0446	0.0492	0.0466	0.0518	0.0518	0.0568	0.0550	0.0726	0.0490	0.0512	0.0496	0.0496
	60%	0.0468	0.0476	0.0464	0.0500	0.0468	0.0484	0.0322	0.0358	0.0422	0.0396	0.0486	0.0460	0.0496	0.0496	0.0452	0.0430	0.0808	0.0570	0.0496	0.0466	0.0492
(20, 50)	0%	0.0476	0.0430	0.0440	0.0582	0.0430	0.0494	0.0340	0.0490	0.0398	0.0446	0.0378	0.0388	0.0502	0.0506	0.0538	0.0526	0.0414	0.0220	0.0448	0.0484	0.0496
	20%	0.0454	0.0432	0.0414	0.0542	0.0420	0.0468	0.0328	0.0500	0.0440	0.0500	0.0402	0.0404	0.0486	0.0492	0.0522	0.0484	0.0502	0.0306	0.0436	0.0398	0.0414
	40%	0.0472	0.0484	0.0452	0.0566	0.0460	0.0512	0.0382	0.0458	0.0490	0.0530	0.0450	0.0434	0.0504	0.0506	0.0524	0.0504	0.0770	0.0524	0.0494	0.0448	0.0452
	60%	0.0480	0.0474	0.0480	0.0526	0.0460	0.0512	0.0396	0.0372	0.0508	0.0540	0.0456	0.0458	0.0484	0.0484	0.0530	0.0490	0.0868	0.0606	0.0520	0.0458	0.0496
(40, 80)	0%	0.0534	0.0434	0.0464	0.0638	0.0434	0.0538	0.0434	0.0494	0.0410	0.0438	0.0442	0.0446	0.0562	0.0566	0.0560	0.0590	0.0412	0.0228	0.0480	0.0516	0.0476
	20%	0.0526	0.0432	0.0470	0.0594	0.0436	0.0534	0.0408	0.0492	0.0418	0.0454	0.0444	0.0440	0.0540	0.0542	0.0576	0.0534	0.0476	0.0288	0.0494	0.0488	0.0460
	40%	0.0498	0.0458	0.0452	0.0616	0.0460	0.0544	0.0416	0.0482	0.0448	0.0468	0.0462	0.0470	0.0550	0.0550	0.0574	0.0500	0.0652	0.0418	0.0518	0.0500	0.0528
	60%	0.0492	0.0494	0.0488	0.0528	0.0486	0.0518	0.0418	0.0388	0.0512	0.0530	0.0494	0.0490	0.0484	0.0484	0.0488	0.0482	0.0654	0.0430	0.0534	0.0480	0.0490
(50, 100)	0%	0.0538	0.0492	0.0520	0.0564	0.0492	0.0520	0.0456	0.0530	0.0484	0.0474	0.0454	0.0456	0.0554	0.0552	0.0552	0.0522	0.0398	0.0254	0.0518	0.0508	0.0556
	20%	0.0502	0.0520	0.0508	0.0554	0.0516	0.0540	0.0486	0.0532	0.0482	0.0496	0.0476	0.0486	0.0518	0.0516	0.0538	0.0552	0.0460	0.0280	0.0544	0.0554	0.0566
	40%	0.0544	0.0562	0.0534	0.0516	0.0536	0.0500	0.0460	0.0532	0.0546	0.0538	0.0518	0.0520	0.0536	0.0536	0.0544	0.0562	0.0610	0.0388	0.0502	0.0546	0.0530
	60%	0.0516	0.0548	0.0530	0.0500	0.0518	0.0492	0.0464	0.0420	0.0558	0.0572	0.0540	0.0524	0.0496	0.0496	0.0528	0.0512	0.0664	0.0414	0.0526	0.0568	0.0548

## Applications

In this section, we apply the above testing procedures to three real data examples that correspond to Situations 2–4. The first example consists of data from kidney dialysis patients, which are described in detail by Klein and Moeschberge [31]. The second consists of data regarding breast cancer patients obtained from the Surveillance, Epidemiology, and End Results (SEER) database. The last example concerns the comparison of two types of treatments for gastric cancer [36].

### Example 1 Kidney dialysis data

**Figure 2:**
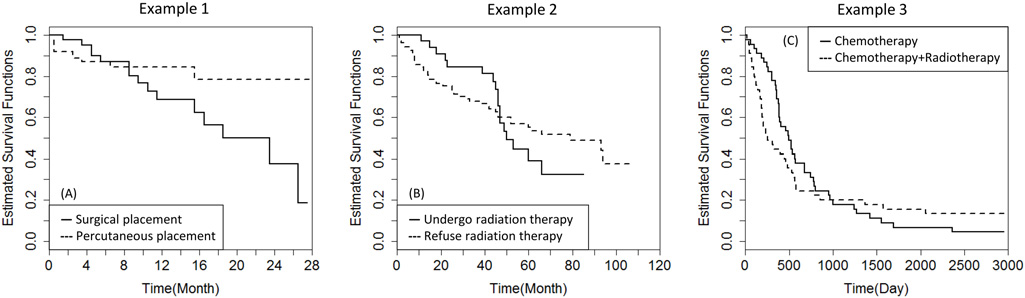
Estimated survival functions for Example 1, 2, and 3.

The kidney-dialysis trial was designed to assess the time to the first exit-site infection (in months) in patients with renal insufficiency. In 43 patients, the catheter was surgically implanted, whereas in 76 patients, the catheter was percutaneously placed. Catheter failure was the primary reason for censoring. There were 27 patients censored in the first group and 65 in the second, corresponding to censoring rates of 62.79% and 85.53%, respectively. As is apparent in Fig. 2A, the two survival curves cross at an early time, and there is a clear difference at later times. Moreover, a test of the proportional-hazards assumption [37] indicated that the assumption is indeed violated in this case (*χ* ^2^ = 8.696, *P* = 0.003). The relevant statistics from various tests and the corresponding *P*-values are listed in Table 6. The conventional LR, G10, GW and TW tests offer relatively low power in this situation because the positive differences are cancelled out by the negative differences, leaving them unable to detect the overall differences. As Table 2 indicates, the RY, CVM1, CVM2, WKM and MKM tests are unstable in the case of heavy censoring. As a result, the above tests fail to identify significant differences between the two groups. However, the G01, SHL1, SHL2, SHL3, MKS, LW, LX, TS, NY1 and NY2 tests yield significant results, and this finding is consistent with the simulation results.

**Table 6:** The results of various procedures for three Examples.

**Methods**	**Example 1**		**Example 2**		**Example 3**	
	**Statistic**	***P-value***	**Statistic**	***P-value***	**Statistic**	***P-value***
LR	1.5904	0.1117	0.2409	0.8096	0.4745	0.6351
GW	-0.0456	0.9636	-0.8569	0.3915	-1.9909	0.0465*
TW	0.6346	0.5257	-0.3562	0.7217	-1.3795	0.1677
G01	3.1093	0.0019*	2.0784	0.0377*	1.4338	0.1516
G10	1.1775	0.2390	-0.3968	0.6915	-1.9909	0.0465*
G11	3.1359	0.0017*	1.7672	0.0772	0.1176	0.9064
RY	1.5904	0.2235	1.5888	0.2242	2.1998	0.0556
MKS	1.3612	0.0294*	0.8705	0.3734	1.7078	0.0059*
CVM1	0.5763	0.2595	1.0477	0.0700	1.9586	0.0345*
CVM2	0.1127	0.1890	0.2344	0.0110*	0.4394	0.0105*
WKM	0.8789	0.3795	-0.7268	0.4673	-0.0396	0.9684
MKM	2.0457	0.0652	1.3904	0.2353	2.4496	0.0315*
SHL1	2.1434	0.0189*	0.8408	0.3655	0.2785	0.7524
SHL2	2.1434	0.0189*	1.2376	0.1835	1.7124	0.0527
SHL3	3.1093	0.0035*	2.0784	0.0627	1.9909	0.0830
LW	2.2516	0.0243*	0.6986	0.4848	1.0829	0.2788
LX1	2.3334	0.0098*	1.7673	0.0386*	1.0371	0.1498
LX2	2.3334	0.0196*	1.7673	0.0772	1.0371	0.2997
TS	NA	0.0273*	NA	0.0253*	NA	0.0253*
NY1	12.9680	0.0120*	12.7881	0.0110*	13.5879	0.0095*
NY2	10.2554	0.0095*	11.9332	0.0070*	13.4548	0.0030*

* Statistical results with *P*<0.05; TS test did not provide the final statistic.

### Example 2 Breast cancer data from SEER

The Surveillance, Epidemiology, End Results (SEER) Program collects data concerning cancer cases in various locations and from sources throughout the United States. We extracted the data regarding 144 black patients who were first diagnosed with breast cancer after the year 2000. Among these patients, we focus only on the comparison between the following two groups: Group 1 consists of 35 patients who underwent radiation therapy as their first course of treatment, and group 2 consists of 109 patients whose guardians refused radiation therapy. The censoring rates of the two groups were 51.43% and 57.80%, respectively. As Fig. 2B illustrates, the two survival curves cross each other near *S*(*t*)=0.6. Meanwhile, a test of the proportional-hazards assumption yielded a result of *χ* ^2^ = 9.470, *P* =0.002. From Fig. 2B, it is evident that radiation therapy could improve a patient’s survival rate if administered in the early period; however, it could not provide long-term benefits, as patients in group 2 survived longer than did those in group 1 after 50 months. The median survival time of each group was 50 months and 79 months, respectively. As shown in Table 6, the G01, CVM2, LX1, TS, NY1 and NY2 tests conclude that the treatment effects in the two groups are significantly different. Because Fig. 2B is analogous to the configurations observed for Situation 3, the result is also comparable.

### Example 3 Gastric cancer data

Stablein and Koutrouvelis [36] have compared two types of therapies (chemotherapy versus chemotherapy combined with radiotherapy) in the treatment of locally unresectable gastric cancer. In this study, 45 patients were each randomly allocated to one of two groups, with censoring rates of 4.44% and 13.33%, respectively. Fig. 2C presents the two survival functions, which cross at approximately *S*(*t*) = 0.2, and the corresponding test indicated a severe violation of the proportional-hazards assumption (*χ* ^2^ = 13.127, *P* < 0.001). As observed in Table 6, the G10 and GW tests are most sensitive to early differences. Moreover, the MKS, CVM1, CVM2, MKM, TS, NY1 and NY2 tests, which demonstrated powers of over 80% in the corresponding simulation scenario, also yielded significant results, as expected. During the initial 1000 days, the use of chemotherapy resulted in a superior survival rate, whereas chemotherapy combined with radiation therapy was associated with an increased number of early deaths, which may be attributable to the progression of tumors within the radiation field or to nutritional and hematological complications. However, chemotherapy combined with radiation therapy appeared to offer better prospects for long-term survival during the late follow-up period.

## Discussion

In clinical research, the violation of the proportional-hazards assumption is often encountered, particularly when two survival curves cross each other. In this paper, we considered a number of tests for the comparison of two survival distributions and investigated their performances for various sample sizes and censoring rates. The objective of our study was to choose a statistical inference method that is appropriate for use when survival curves cross. Because survival functions are more common and intuitive than hazard functions when investigating the survival differences between two groups, simulations were performed for situations in which the survival distributions crossed at early, middle and late times. The simulation results demonstrated that the NY1, NY2 and TS tests had a robust power under various situations of crossing survival curves with reasonable type I error rates. The study confirmed that the location of the crossing point may affect the discrimination power of the test statistic employed. Pepe and Fleming [23] have claimed that the WKM test performs much better than does the LR test in the case of crossing hazards; however, the simulation results confirmed this conclusion only for the late crossing of the survival curves (Situation 4). Moreover, the simulation results also suggested that when the two survival curves cross at an early time (Situation 2) and when the censoring rate is low (≤20%), the LR test maintains a reasonable power of greater than 80%, which is consistent with the results of Tubert-Bitter [27].

A simulation of time-to-event data was performed using a randomly censored model. To ensure that the average censoring rates in each tested group were consistent with the specified censoring rates, it was necessary for the uniform parameters of the two groups to be unequal. In Situaion 4, as the censoring rate was increased from 20% to 40%, the powers of several weighted log-rank tests (LR, G10, GW and TW) improved, as observed by Tubert-Bitter [27]. Such an increase in the censoring rate may lead to a shortening of the maximum length of the follow-up, which reduces the cancellation of positive differences by negative ones, thereby causing the powers of the four conventional tests to unexpectedly increase. However, for the methods specifically designed to address crossing hazards, such as the MKS, TS, NY1 and NY2 tests, their powers gradually decrease as the censoring rate increases. When the censoring rate reaches 60%, the maximum follow-up length (*t* = 2.5) is below the theoretical crossing point (*t* = 5.482). In this particular situation, late crossings cannot be observed for some samples. In a test of the proportional-hazards assumption [37], we found that in 5000 iterations, only 551 samples (11.02%) were non-proportional, whereas 88.98% of the samples satisfied the proportional-hazards assumption. This significant difference in statistics might explain the sudden increase in power of some tests when the censoring rate reached 60%. Moreover, for higher censoring rates, the estimated rate of type I errors for the LX test is quite unstable. Lin and Xu [1] have suggested that the value of the correlation coefficient *ρ_j, j’_* might influence the type I error rate; therefore, these authors set the correlation coefficient to 0.5 to maintain a reasonable type I error rate and a high power. However, as is demonstrated by Tables 1–5 and by the sensitivity analysis presented in S1 Text, better performance can be achieved if the value of *ρ_j, j’_* is varied based on the specific conditions of the problem.

When comparing the limitations and strengths of the various methods, we first find that the performances of the WKM and weighted log-rank tests (LR, G01, G10, G11, GW and TW) are subject to the choice of weight functions. A misspecification of the weights can lead to a substantial loss of power. Moreover, the creation of multiple comparison problems and the inflation of the overall type I error rate might cause difficulties for the user. Second, some modified tests such as the SHL1, SHL2 and SHL3 tests are more likely to be influenced by a high censoring rate. Third, the LW test exhibits lower power in the cases of heavy censoring rates and unbalanced design, and the estimated type I error rate of the LX test often deviates from the nominal level of 0.05. Both methods require much future research. By contrast, as evidenced by the performances of all tests in various scenarios, the NY1, NY2 and TS tests exhibit higher power and greater stability than the other methods even for heavy censoring rates, and the type I error rates for these tests are also close to the nominal level. Because the TS test is not subject to weight functions, it is a particularly convenient method for addressing all possible alternatives. In conclusion, for the testing of the equality of two survival distributions, the NY1, NY2 and TS tests are the most appropriate and robust of all tests evaluated here, especially in the case of two crossing survival curves.

All testing approaches mentioned above are intended for overall hypothesis testing. However, sometimes the greatest interest is focused on which treatment can provide the better short- or long-term survival rate, even when the survival curves cross. Klein et al. [38] have reviewed a number of methods for comparing two survival curves at a fixed point in time. Moreover, Logan et al. [39] have considered several methods for the long-term survival comparison after a pre-specified time point.

For non-proportional hazards problem, Liu et al. [3] have highlighted the relationship between the crossing of survival functions and the crossing of hazard rates: “*if two continuous survival functions cross each other once*, *then the corresponding hazard rates must cross each other at least once*. *However*, *it is possible that survival functions do not cross*, *but their hazard rates cross very often*.” Overall, methods based on hazard rates are more sensitive in terms of capturing the differences and contain more information. Therefore, analyzing the survival curves is not sufficient. In clinical practice, it is necessary to explore the crossing time point of two crossing hazard functions at which the new treatment effect may change reversely, in comparison with the standard treatment. Cheng et al. [40] have developed a nonparametric procedure for constructing the confidence intervals for the first crossing point of two hazard functions. In addition to the patients’ survival data, longitudinal data regarding some time-dependent covariates should be considered to assess the effect of treatment. Recently, Park and Qiu [41] have proposed a joint modeling procedure for analyzing both the survival and longitudinal data with crossing hazard rate functions. In summary, depending on the particular conditions of the study, researchers may wish to consider overall hypothesis testing in combination with the various methods for a more reliable and comprehensive statistical analysis of the survival data.

## Supporting Information

S1 Table Hypothesis tests for two-sample survival data. (PDF)

S1 Text Factors affected the power and type I error rate of Lin-Xu test. The relations among the censoring rate, the correlation coefficient and the power and type I error rate for N1 = N2 = 50 with 5000 iterations for LX1 and LX2 tests. In Fig. 1, it can be observed that the power decreases as the censoring rate and correlation coefficient increase. In Fig. 2, when the censoring rate is specified, the type I error rate decreases as the correlation coefficient increases.
(PDF)
