# Supplementary material for: Statistical Inference Methods for Two Crossing Survival Curves: A Comparison of Methods
Source: PLoS One. 2015 Jan 23;10(1):e0116774. doi: 10.1371/journal.pone.0116774 (PMC4304842; doi:10.1371/journal.pone.0116774)
Supplement: S1 Table — (PDF) [file pone.0116774.s001.pdf]

Table S1. Hypothesis tests for two-sample survival data

No.	Name	Test statistic	Notations
1	Weighted Log-rank	$Z = \frac{[\sum_{j=1}^{\tau} W(t_j)(d_{1j} - n_{1j}\left(\frac{d_j}{n_j}\right))]^2}{\sum_{j=1}^{\tau} W(t_j)^2 \left(\frac{n_{1j}}{n_j}\right)\left(1-\frac{n_{1j}}{n_j}\right)\left(\frac{n_j-d_j}{n_j-1}\right) d_j} \sim \chi^2(1)$	$W(t_j)=1$, Logrank test (LR);<br>$W(t_j)=n_j$, Gehan-Wilcoxon test (GW);<br>$W(t_j)=\sqrt{n_j}$, Tarone-Ware test (TW);<br>$W(t_j)=[S(t_{j-1})]^{\rho}[1-S(t_{j-1})]^{\gamma}$ with $\rho\ge0$, $\gamma\ge0$.<br>Fleming-Harrington test ($G^{\rho,\gamma}$),<br>where d_{ij} is the number of events in the ith group (i=1, 2) out of n_{ij} individuals (at risk) at time t_j and $d_j=d_{1j}+d_{2j}$, $n_j=n_{1j}+n_{2j}$.
2	Renyi(RY)	$Q = \sup\{\lvert\tilde{Z}(t_j)\rvert, t_j \le \tau\} / \sigma(\tau)$	$\tilde{Z}(t_j) = \sum_{t_k \le t_j} W(t_k)\left[d_{1k} - n_{1k}\left(\frac{d_k}{n_k}\right)\right], j=1,2,...\tau,$<br>$\sigma^2(\tau) = \sum_{t_k\le\tau} W(t_k)^2 \left(\frac{n_{1k}}{n_k}\right)\left(\frac{n_{2k}}{n_k}\right)\left(\frac{n_k-d_k}{n_k-1}\right) d_k$
3	Modified Kolmogorov-Smirnov(MKS)	$y = \sup_{0\le t\le T} \lvert Y(t)\rvert$	$Y_{N_1,N_2}(t) = \frac{1}{2}\{\hat{S}_1(t)+\hat{S}_2(t)\}\int_0^t \{\frac{N_1\hat{C}_1(s-)N_2\hat{C}_2(s-)}{N_1\hat{C}_1(s-)+N_2\hat{C}_2(s-)}\}^{\frac{1}{2}} \times I_{\{N_1(s)N(s)>0\}} d\{\hat{H}_1(s)-\hat{H}_2(s)\}$<br>$T = \max\{t_j : N_1(t_j)N_2(t_j) > 0\}$
4	Cramer-von Mises(CVM)	$\int_0^{\tau}[\int_0^{t} K(s) d\{\hat{A}(s) - \hat{A}_0^*(s)\}]^2 dH(s)$	CVM1 statistic uses untransformed process, which is asymptotic to a standard Brownian motion process, another CVM2 statistic converges to a Brownian bridge process

5	Weighted Kaplan-Meier (WKM)	$V_{wkm} = \sqrt{\frac{N_1N_2}{N_1+N_2}}\int_0^{T_m}\tilde{w}(t)[\widehat{S}_2(t)-\widehat{S}_1(t)]dt$ $\hat{\sigma}^2_{wkm} = -\int_0^{T_m}\left\{\int_t^{T_m}\left[\tilde{w}(s)\hat{S}(s)\right]ds\right\}^2 \frac{N_1\widehat{C}_1(t-)+N_2\widehat{C}_2(t-)}{(N_1+N_2)\widehat{C}_1(t-)\widehat{C}_2(t-)}\frac{d\hat{S}(t)}{\hat{S}(t)\hat{S}(t-)}$	$T_m = \sup\{t:\min\{\widehat{C}_1(t),\widehat{C}_2(t),\widehat{S}_1(t),\widehat{S}_2(t)\}>0\}$ $\tilde{w}(t)=\frac{(N_1+N_2)\widehat{C}_1(t-)\widehat{C}_2(t-)}{N_1\widehat{C}_1(t-)+N_2\widehat{C}_2(t-)}$
6	Maximum Weighted Kaplan-Meier (MKM)	$\tilde{V} = \max\lvert\tilde{V}_\ell\rvert$	$V_l = \sqrt{\frac{N_1N_2}{N_1+N_2}}\int_0^{\tau} f\{\hat{S}(t-)\}\tilde{w}(t)[\hat{S}_1(t)-\hat{S}_2(t)]dt$ $f\{S(t-)\}=[S(t-)]^\rho[1-S(t-)]^\gamma, \rho=0,1,2;\gamma=0,1,2$ $\tilde{V}_\ell = V_\ell/(\hat{\sigma}_{\ell,\ell})^{1/2}, \ell=1,\ldots,m(m=9)$
7	Lee's versatile test(SHL)	$SHL1: \lvert Z_1+Z_2\rvert/2$ $SHL2: (\lvert Z_1\rvert+\lvert Z_2\rvert)/2$ $SHL3: \max(\lvert Z_1\rvert,\lvert Z_2\rvert)$	Z_1 and Z_2 are calculated as Z_1 with weight function ρ=0, γ=1; Z_2 with weight function ρ=1, γ=0;
8	Lin and Wang's test(LW)	$T^* = \frac{\Delta - E(\Delta)}{\sqrt{Var(\Delta)}} \sim N(0,1)$	$\Delta = \sum_{j=1}^{\tau}\left[d_{1j}-E(d_{1j})\right]^2$ $E(\Delta)=\sum_{j=1}^{\tau}\frac{n_{1j}n_{2j}d_j(n_j-d_j)}{n_j^2(n_j-1)}$ $Var(\Delta)=\sum_{j=1}^{\tau}\{E(d_{1j}^4)-4E(d_{1j}^3)E(d_{1j})+6E(d_{1j}^2)(E(d_{1j}))^2-3(E(d_{1j}))^4-(Var(d_{1j}))^2\}$

9	Lin and Xu's test (LX)	$\Delta^* = \frac{\Delta - \widehat{E}(\Delta)}{\sqrt{\widehat{Var}(\Delta)}} \sim N(0,1)$	$\Delta = \sum_{t_j<\tau} \vert \widehat{S}_1(t_j) - \widehat{S}_2(t_j) \vert (t_{j+1} - t_j)$<br>$\widehat{E}(\Delta) = \sum_{j\vert t_j<\tau} \{\frac{2}{\pi}[\hat{\sigma}^2_{S_1}(t_j) + \hat{\sigma}^2_{S_2}(t_j)]\}^{1/2}(t_{j+1} - t_j)$<br>$\widehat{Var}(\Delta) = \sum_{j\vert t_j<\tau} (t_{j+1} - t_j)^2(1-\frac{2}{\pi})[\hat{\sigma}^2_{S_1}(t_j) + \hat{\sigma}^2_{S_2}(t_j)] + \sum_{j<j'\vert t_j,t_{j'}<\tau} 2\rho_{j,j'}(t_{j+1} - t_j)(t_{j'+1} - t_{j'})(1-\frac{2}{\pi})$<br>$\times\{[\hat{\sigma}^2_{S_1}(t_j) + \hat{\sigma}^2_{S_2}(t_j)][\hat{\sigma}^2_{S_1}(t_{j'}) + \hat{\sigma}^2_{S_2}(t_{j'})]\}^{1/2}$
10	Two-stage(TS)	$V = \sup_{\tau_\varepsilon \le m \le \tau - \tau_\varepsilon} (V_m)$	$V_m = \sum_{j=1}^{\tau} w_j^{(m)}(d_{1j} - n_{1j}\frac{d_j}{n_j}) \Big/ \sqrt{\sum_{j=1}^{\tau} (w_j^{(m)})^2 \left(\frac{n_{1j}}{n_j}\right)\left(\frac{n_{2j}}{n_j}\right)\left(\frac{n_j - d_j}{n_j - 1}\right) d_j}$<br>$w_j^{(m)} = \begin{cases} -1, & j = 1,2...m \\ c_m, & j = m+1,...\tau \end{cases}$
11	Adaptive Neyman's Smooth tests (NY)	$T_d = U(\tau)^{\mathrm{T}}\hat{\sigma}(\tau)^{-}U(\tau)$<br>$T_s = \max\{V_C(\tau)^{\mathrm{T}}\sigma_{CC}(\tau)^{-1}V_C(\tau) : C \in \zeta, \vert C \vert = d^*\}$	T_d : fixed-dimensional test(NY1)<br>T_s : data-driven test(NY2)<br>$V_C(\tau)$ and $\hat{\sigma}_{CC}(\tau)$ are the subvector and submatrix of $V(\tau)$ and $\sigma(\tau)$ corresponding to the subset *C*. Schwarz's criterion is given as $S = \arg\max_{C\in\zeta}\{T_C - \vert C \vert \log n\}$.
