# Supplementary material for: Statistical Inference Methods for Two Crossing Survival Curves: A Comparison of Methods
Source: PLoS One. 2015 Jan 23;10(1):e0116774. doi: 10.1371/journal.pone.0116774 (PMC4304842; doi:10.1371/journal.pone.0116774)
Supplement: S1 Text — The relations among the censoring rate, the correlation coefficient and the power and type I error rate for N1 = N2 = 50 with 5000 iterations for LX1 and LX2 tests. In Fig. 1, it can be observed that the power decreases as the censoring rate and correlation coefficient increase. In Fig. 2, when the censoring rate is specified, the type I error rate decreases as the correlation coefficient increases. (PDF) [file pone.0116774.s002.pdf]

Text S1. Factors affected the power and type I error of Lin-Xu test

As Lin and Xu [1] have mentioned in their study, the test statistic depends on the correlation coefficient $\rho_{j,j'}$; these authors set $\rho_{j,j'}$=0.5 to ensure a reasonable type I error rate and reasonable power. However, as Table 5 in the main text demonstrates, the type I error rates of the LX1 and LX2 tests both deviate from the nominal level. Therefore, we performed Monte Carlo simulations to study the relations among the censoring rate, the correlation coefficient and the power and type I error rates for N_1=N_2=50 with 5000 iterations. In Figure 1, it can be observed that the power decreases as the censoring rate and correlation coefficient increase. In Figure 2, when the censoring rate is specified, the type I error rate decreases as the correlation coefficient increases.

Reference

1. Lin X, Xu Q. (2010) A new method for the comparison of survival distributions. Pharm Stat 9(1): 67-76.

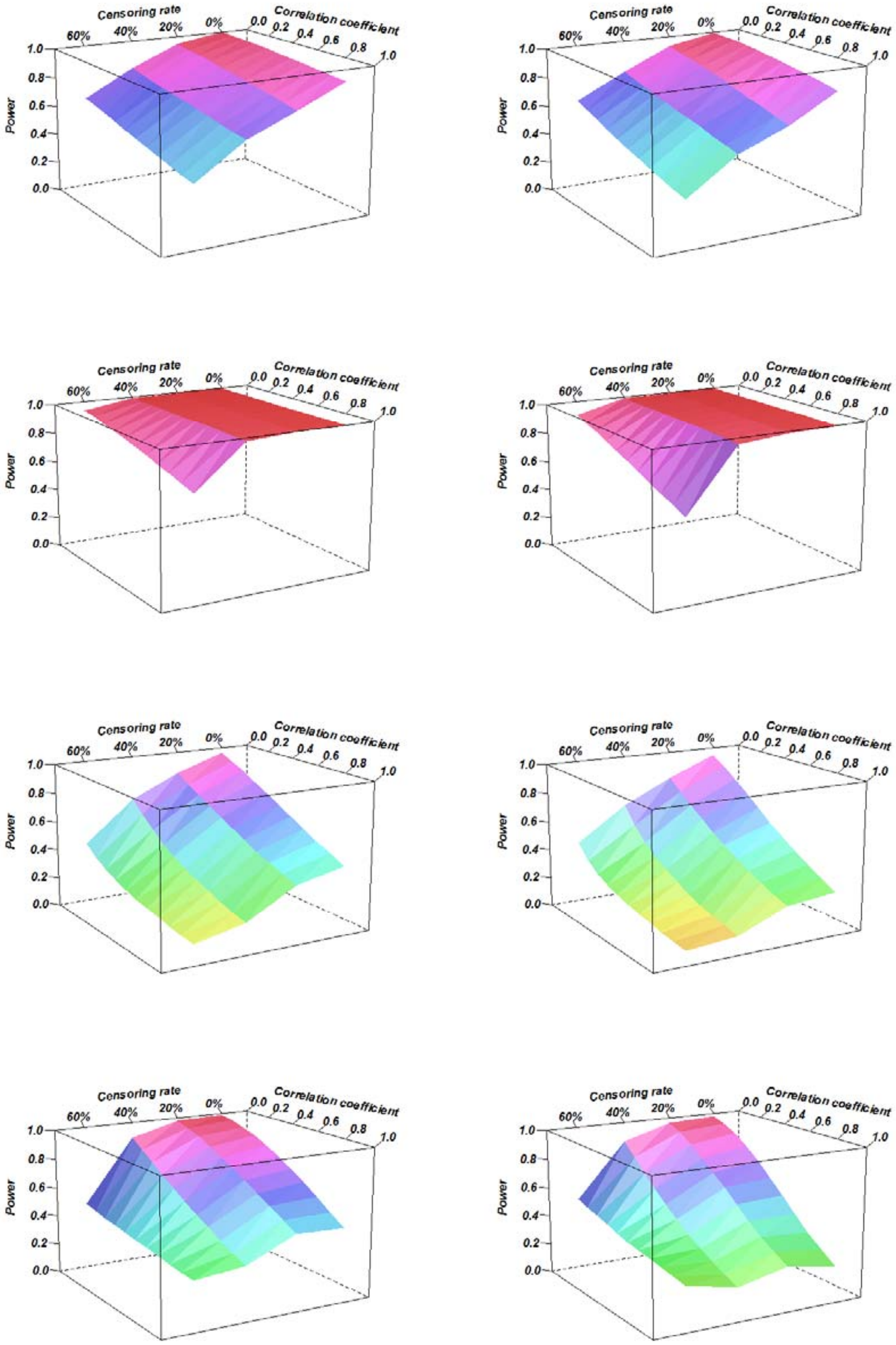

Figure 1. The relationship among censoring rate, correlation coefficient and power under N_1=N_2=50 with 5000 replications. From top to bottom, the figures denote the results of Situation 1~4 with LX1 (left) and LX2 (right) tests.

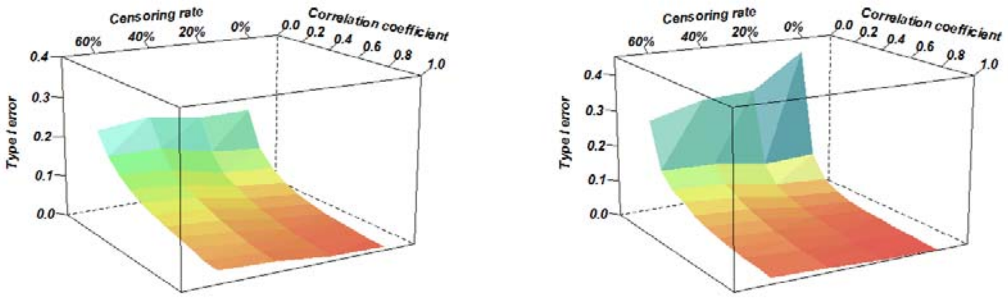

Figure 2. The relationship among censoring rate, correlation coefficient and type I error rate under N_1=N_2=50 with 5000 replications.
